# Genomic comparisons and phylogenetic analysis of mastitis-related staphylococci with a focus on adhesion, biofilm, and related regulatory genes

**DOI:** 10.1038/s41598-021-96842-2

**Published:** 2021-08-30

**Authors:** Lucas José Luduverio Pizauro, Camila Chioda de Almeida, Saura Rodrigues Silva, Janet I. MacInnes, Andrew M. Kropinski, Luiz Francisco Zafalon, Fernando Antônio de Avila, Alessandro de Mello Varani

**Affiliations:** 1 Department of Technology, Sao Paulo State University, Jaboticabal, Sao Paulo Brazil; 2 Department of Microbiology, Sao Paulo State University, Jaboticabal, Sao Paulo Brazil; 3 Department of Pathobiology, Ontario Veterinary College, University of Guelph, Guelph, ON Canada; 4 Brazilian Agricultural Research Corporation (EMBRAPA), Embrapa Southeast Livestock, Sao Carlos, Sao Paulo Brazil

**Keywords:** Comparative genomics, Microbiology

## Abstract

Mastitis is a common and costly disease on dairy farms, commonly caused by *Staphylococcus* spp. though the various species are associated with different clinical outcomes. In the current study, we performed genomic analyses to determine the prevalence of adhesion, biofilm, and related regulatory genes in 478 staphylococcal species isolated from clinical and subclinical mastitis cases deposited in public databases. The most prevalent adhesin genes (*ebpS*, *atl*, *pls*, *sasH* and *sasF*) were found in both clinical and subclinical isolates. However, the *ebpS* gene was absent in subclinical isolates of *Staphylococcus arlettae, S. succinus, S. sciuri, S. equorun, S. galinarum,* and *S. saprophyticus*. In contrast, the *coa*, *eap*, *emp, efb,* and *vWbp* genes were present more frequently in clinical (vs. subclincal) mastitis isolates and were highly correlated with the presence of the biofim operon (*icaABCD*) and its transcriptional regulator, *icaR*. Co-phylogenetic analyses suggested that many of these adhesins, biofilm, and associated regulatory genes could have been horizontally disseminated between clinical and subclinical isolates. Our results further suggest that several adhesins, biofilm, and related regulatory genes, which have been overlooked in previous studies, may be of use for virulence profiling of mastitis-related *Staphylococcus* strains or as potential targets for vaccine development.

## Introduction

Bovine mastitis is one of the costliest diseases seen on dairy farms, with an estimated global loss of 19.7 to 32 billion US$ due to reduced milk production and withdrawal periods related to antibiotic usage^1^. Mastitis may also cause death directly or lead to the slaughter of chronically infected animals^1^. *S. aureus* is generally considered the most important cause of both clinical and subclinical mastitis, while coagulase-negative staphylococci or non-aureus *Staphylococcus* spp. are thought to be of lesser importance or as opportunistic pathogens^2^. A higher prevalence of subclinical versus clinical infections has been reported^3^. Moreover, the prevalence of subclinical mastitis is likely to be underestimated due to the lack of obvious signs except for changes in milk quantity and quality (which can only be detected by specific tests such as the California Mastitis Test and by somatic cell counting)^1^. It is generally believed that the *Staphylococcus* spp. isolates associated with chronic infections are different from those that cause acute infections and are more likely to be transmitted and persist in the herd due to better host adaptation and the absence of overt clinical signs^1,2^.

Attachment and colonization are crucial in the development of mastitis. In *Staphylococcus* spp., factors such as the fibronectin-binding proteins (*fnbA* and *fnbB*), elastin binding proteins (*ebpS*), clumping factors (*clfA* and *clfB*), and collagen-binding protein (*cna*) play important roles in binding to host cells, colonization, and invasion^2^. The *pls* gene, which encodes the plasmin-sensitive protein, also has an important role in bacterial adherence^4^. The surface proteins encoded by *sasH* and *sasF*, play a significant role in virulence because they bind to host extracellular matrix and plasma components. Recently, they have been reported to be prevalent adhesins in genomes of *Staphylococcus* spp. isolates from cattle^5^. In addition, the *ica* genes, which are associated with the synthesis of polysaccharide intercellular adhesin (PIA), are thought to play a crucial role in biofilm development in these bacteria.

Molecular epidemiology-based methods such as specific PCR assays, MLST, and PFGE have been used to analyze the genetic diversity and virulence factors and to track the dissemination of *Staphylococcus* spp. infections, but they have limitations^1^. Accordingly, the current work aimed to ascertain the prevalence of adhesion and biofilm genes by investigating whole genome sequences of *Staphylococcus* spp. from clinical and subclinical mastitis cases and evaluate the phylogenetic relationship of these isolates and determine if any adhesin or biofilm genes associated with acute bovine/bubaline mastitis.

## Results

### Assessment of clinical and subclinical mastitis Staphylococcus spp. isolates

**Figure 1:**
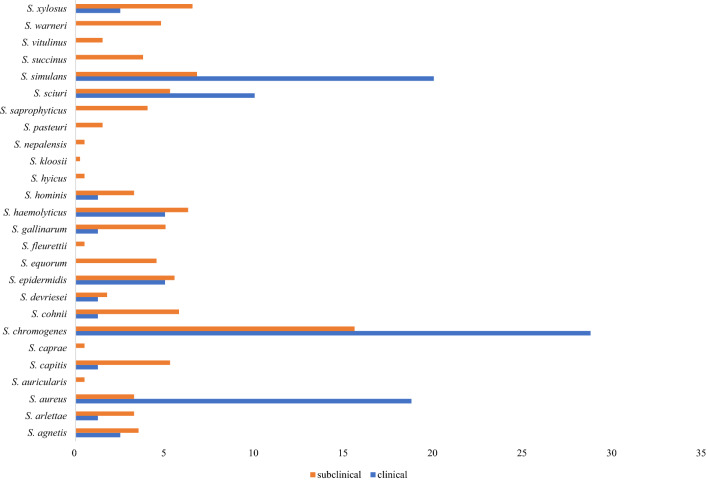
Frequency of staphylococcal species associated with clinical and subclinical mastitis.

*Staphylococcus chromogenes* (28.7%), *S. simulans* (20.0%). *S. aureus* (18.7%) and the *S. sciuri* (10.0%) were the most prevalent clinical mastitis species deposited in the NCBI GenBank database. The most frequent staphylococcal species associated with subclinical mastitis were *S. chromogenes* (15.6%), *S. simulans* (6.8%), *S. xylosus* (6.5%), *S. haemolyticus* (6.3%), *S. cohnii* (5.8%), *S. epidermidis* (5.5%), *S. capitis* (5.3%), *S. sciuri* (5.3%) (Table 1, Fig. 1).

**Table 1:** Relative and absolute frequency of staphylococcal species obtained from complete genome of staphylococci associated with bovine/bubaline mastitis.

Species	Mastitis	
	Clinical	Subclinical
*S. agnetis*	2.50% (2)	3.52% (14)
*S. arlettae*	1.25% (1)	3.27% (13)
*S. aureus*	18.7% (15)	3.27% (13)
*S. auricularis*	0.00% (0)	0.50% (2)
*S. capitis*	1.25% (1)	5.28% (21)
*S. caprae*	0.00% (0)	0.50% (2)
*S. chromogenes*	28.7% (23)	15.5% (62)
*S. cohnii*	1.25% (1)	5.78% (23)
*S. devriesei*	1.25% (1)	1.76% (7)
*S. epidermidis*	5.00% (4)	5.53% (22)
*S. equorum*	0.00% (0)	4.52% (18)
*S. fleurettii*	0.00% (0)	0.50% (2)
*S. gallinarum*	1.25% (1)	5.03% (20)
*S. haemolyticus*	5.00% (4)	6.28% (25)
*S. hominis*	1.25% (1)	3.27% (13)
*S. hyicus*	0.00% (0)	0.50% (2)
*S. kloosii*	0.00% (0)	0.25% (1)
*S. nepalensis*	0.00% (0)	0.50% (2)
*S. pasteuri*	0.00% (0)	1.51% (6)
*S. saprophyticus*	0.00% (0)	4.02% (16)
*S. sciuri*	10.0% (8)	5.28% (21)
*S. simulans*	20.0% (16)	6.78% (27)
*S. succinus*	0.00% (0)	3.77% (15)
*S. vitulinus*	0.00% (0)	1.51% (6)
*S. warneri*	0.00% (0)	4.77% (19)
*S. xylosus*	2.50% (2)	6.53% (26)
Total	100% (80)	100% (398)

### Distribution of adhesin, biofilm and regulatory genes

**Figure 2:**
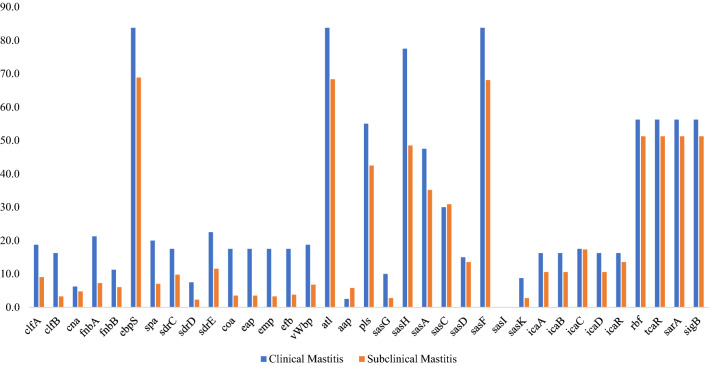
Frequency of adhesin and biofilm-related genes in mastitis-associated staphylococcal isolates.

In the mastitis related staphylococcal genomes analyzed (n = 478) the most prevalent genes associated with adhesion and biofilm formation were: *ebpS* (71.3%), *atl* (70.9%), *sasF* (70.7%), *sasH* (53.3%), *araC* (52.1%), *tcaR* (52.1%), *sarA* (52.1%), *sigB* (52.1%) *pls* (44.6%), *sasA* (37.2%) and *sasC* (30.8%) (Table 2, Fig. 2).

**Table 2:** Relative and absolute frequency of adhesin, biofilm genes, and related regulatory genes of staphylococcal species associated with clinical and subclinical mastitis.

Role	Gene	Mastitis	
		Clinical	Subclinical
Clump factor A	*clfA*	18.7% (15)	9.05% (36)
Clump factor B	*clfB*	16.2% (13)	3.27% (13)
Collagen adhesion	*cna*	6.25% (5)	4.77% (19)
Fibronectin binding protein A	*fnbA*	21.2% (17)	7.29% (29)
Fibronectin binding protein B	*fnbB*	11.2% (9)	6.03% (24)
Elastin binding protein	*ebpS*	83.8% (67)	68.8% (274)
Staphylococcal protein A	*spa*	20.0% (16)	7.04% (28)
Ser-Asp rich fibrinogen-binding protein C	*sdrC*	17.5% (14)	9.80% (39)
Ser-Asp rich fibrinogen-binding protein D	*sdrD*	7.50% (6)	2.26% (9)
Ser-Asp rich fibrinogen-binding protein E	*sdrE*	22.5% (18)	11.5% (46)
Staphylocoagulase	*coa*	17.5% (14)	3.52% (14)
Extracellular adherence protein Eap/Map	*eap*	17.5% (14)	3.52% (14)
Extracellular matrix protein-binding protein	*emp*	17.5% (14)	3.27% (13)
Fibrinogen binding protein	*efb*	17.5% (14)	3.77% (15)
Secreted von Willebrand factor-binding protein	*vWbp*	18.7% (15)	6.78% (27)
Bifunctional autolysin	*atl*	83.8% (67)	68.3% (272)
Accumulation associated protein	*aap*	2.50% (2)	5.78% (23)
Surface protein	*pls*	55.0% (44)	42.4% (169)
Surface protein G	*sasG*	10.0% (8)	2.76% (11)
Surface protein H	*sasH*	77.5% (62)	48.4% (193)
Surface protein A	*sasA*	47.5% (38)	35.1% (140)
Surface protein C	*sasC*	30.0% (24)	30.9% (123)
Surface protein D	*sasD*	15.0% (12)	13.5% (54)
Surface protein F	*sasF*	83.8% (67)	68.0% (271)
Surface protein I	*sasI*	0.00% (0)	0.00% (0)
Surface protein K	*sasK*	8.75% (7)	2.76% (11)
Biofilm operon protein A	*icaA*	16.2% (13)	10.5% (42)
Biofilm operon protein B	*icaB*	16.2% (13)	10.5% (42)
Biofilm operon protein C	*icaC*	17.5% (14)	17.3% (54)
Biofilm operon protein D	*icaD*	16.2% (13)	10.5% (42)
Biofilm negative transcriptional regulator	*icaR*	16.2% (13)	13.5% (54)
regulation of biofilm formation	*rbf*	56.3% (45)	51.2% (204)
Transcriptional regulator involved in teicoplanin susceptibility	*tcaR*	56.3% (45)	51.2% (204)
Transcriptional regulator involved in biofilm formation process	*sarA*	56.3% (45)	51.2% (204)
RNA polymerase sigma factor B	*sigB*	56.3% (45)	51.2% (204)

In strains associated with clinical mastitis, the *ebpS* (83.8%), *atl* (83.8%), *sasF* (83.8%), *sasH* (77.5%), *rbf* (56.3%), *tcaR* (56.3%), *sarA* (56.3%), *sigB* (56.3%), *pls* (55.0%), *sasA* (47.5%), *pls* (37.5%) and *sasC* (30.0%) genes were most frequently detected. The carriage of adhesin/biofilm related genes in isolates associated with subclinical mastitis were less frequent (e.g., *ebpS* (68.8%), *atl* (68.3%), *sasF* (68.1%), *atl* (51.3%), *rbf* (51.3%), *tcaR* (51.3%), *sarA* (51.3%), *sigB* (51.3%) *sasH* (48.5%) *pls* (42.5%), *sasA* (35.2%) and *sasC* (30.9%) (Table 2).

Most of the subclinical isolates of *S. aureus, S. capitis, S. chromogenes, S. cohnii, S. epidermidis, S. haemolyticus, S. warneri* and *S. simulans* had the *ebpS, atl, pls, sasH, sasC* and *sasF* adhesion-associated genes with the *clfB* and *emp* genes found only in *S. aureus* strains. The PIA production operon *icaADBC* and its regulator *icaR* was only present in *S. aureus*, *S. chromogenes*, *S. capitis*, and *S. epidermidis*; most *S. cohnii* and *S. saprophyticus* carried the *icaC* gene. In the clinical isolates, the *icaADBC* operon and *icaR* gene were present only in *S. aureus* isolates. Other biofilm regulatory genes i.e., *rbf*, *tcaR*, *sarA* and *sigB* were found in subclinical isolates of *S. aureus*, *S. chromogenes*, *S. epidermidis* and *S. haemolyticus*, but not in *S. simulans* isolates (Supplementary Table 1).

### Phylogenetic analyses reveal no clear relationship between clinical and subclinical isolates

Analysis of the 16S RNA genes from the genome sequences of the *Staphylococcus* spp. from bovine and buffalo mastitis cases revealed that the clinical and subclinical isolates (n = 478) are present in a wide variety of clades and do not show any clear relationship (Supplementary Fig. 1). The 16S RNA gene phylogeny also indicated that the mastitis related *S. aureus, S. epidermidis,* and *S. capitis* have a close phylogenetic relationship. These species also possess many adhesion genes (avg. no. = 26, 11, and 17 respectively), followed by *S. chromogenes* and *S. warneri* (avg. no. = 9 and 12, respectively). *S. capitis* has a close phylogenetic relationship to the species that are mainly associated with clinical mastitis (*S. aureus* and *S. epidermidis*). *S. chromogenes*, which was implicated in cases of clinical (n = 23/80) and subclinical mastitis (n = 61/398), is most closely related to *S. agnetis* and *S. hyicus* species that were only associated with subclinical mastitis. “Subclinical species” *S. saprophyticus, S. xylosus, S. gallinarum* and *S. arlettae* formed a distinct node with few strains involved in clinical mastitis and with most of these strains not carrying known adhesion/biofilm related genes. The “subclinical species” *S. warneri* and *S. pasteuri* were also phylogenetically related and carried biofilm/adhesion associated genes (n = 35; avg. no. of genes = 12 and 10, respectively). No specific pattern was observed between clinical and subclinical strains based on *ebpS, rbf, sarA, sasH, sigB,* and *tcaR* gene phylogeny (Supplementary Figs. 2–7, respectively). Generally, the clinical and subclinical strains of most species were in the same clade.

The co-phylogenetic analysis suggested that there may be movement of virulence genes by horizontal gene transfer (HGT) in staphylococcal spp. For example, the *ebpS* gene may have moved among clinical and subclinical isolates of *S. simulans, S chromogenes* and *S. aureus* (Supplementary Fig. S8); the *pls* gene among clinical and subclinical isolates of *S. haemolyticus, S. chromogenes* and *S. simulans* (Supplementary Fig. S9); the *rbp* gene among clinical and subclinical isolates of *S. chromogenes, S. aureus* and *S. haemolyticus* (Supplementary Fig. S10), and the *sarA* gene, among clinical and subclinical isolates of *S. chromogenes* and *S. aureus*, respectively (Supplementary Fig. S11). Possible HGT was also observed for the *sasH* gene among *S. aureus*, *S. simulans* and *S. chromogenes* (Supplementary Fig. S12) and the *tcaR* gene among the *S. aureus*, and *S. chromogenes* clinical and subclinical isolates (Supplementary Fig. S13). Although the co-phylogenetic analysis suggested that HGT might be taking place, further investigatation of the association of these virulence genes with mobile genetic elements, such as transposons and *S. aureus* pathogenicity islands (SaPIs), must be carried out to test the HGT hypothesis.

### Data analysis indicates adhesion and biofilm genes exclusively related to clinical isolates

**Figure 3:**
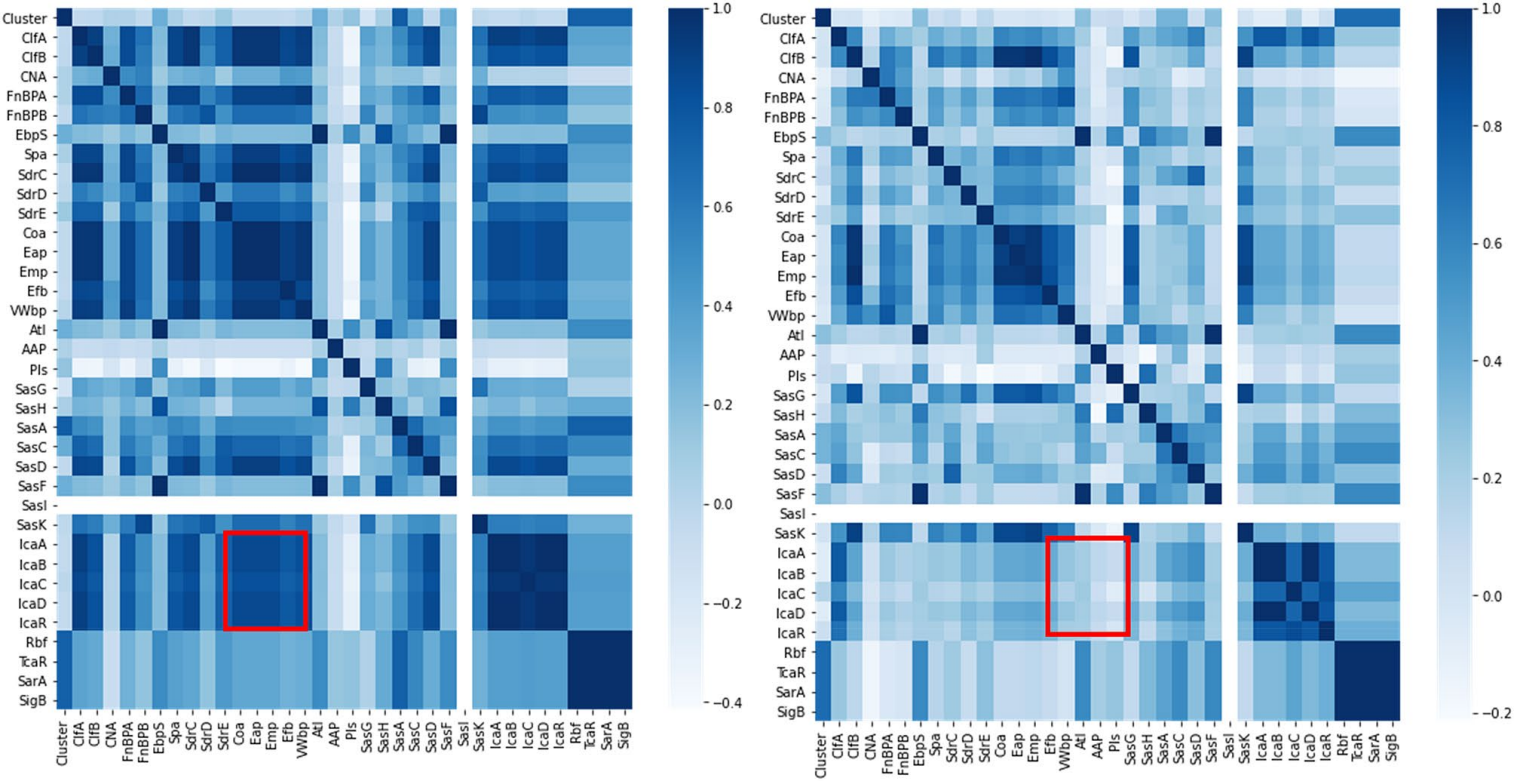
Heat map of adhesion and biofilm genes in clinical and subclinical staphylococcal isolates (Left—Clinical mastitis; Right—Subclinical mastitis). A higher correlation between the presence of the *ica*ABCDR and the *coa*, *eap*,*emp*,*efb* and *vWbp* was found in clinical (vs. subclinical) isolates (red box).

**Figure 4:**
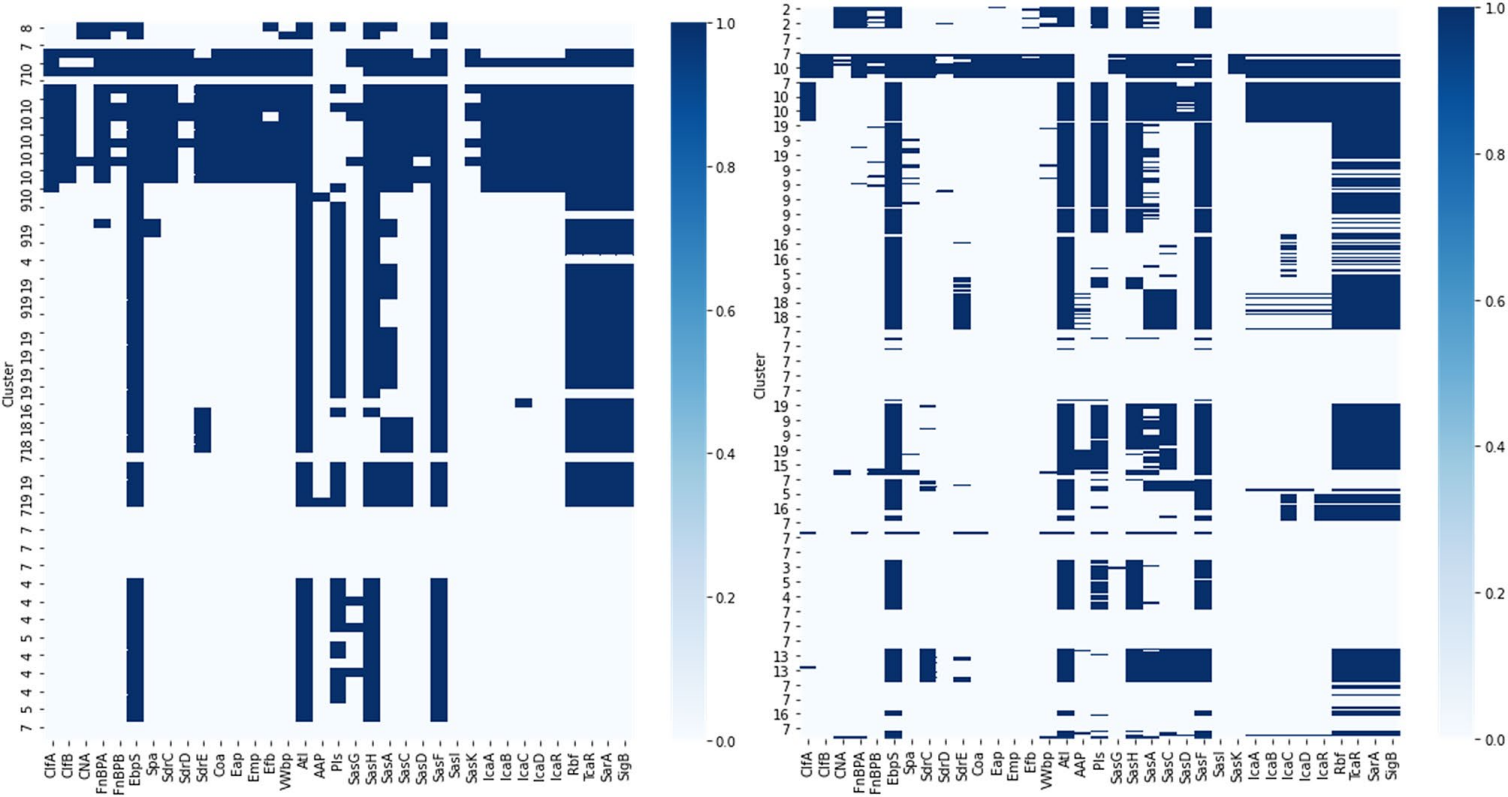
Heat map of the f requency of adhesin and biofilm genes found in *Staphylococcus* spp. clusters associated with clinical and subclinical mastitis (Left—Clinical mastitis; Right—Subclinical mastitis). The *ebpS*, *atl*, *pls*,*sasH*, *sasF*, *tcaR*, *sarA* and *sigB* were most frequently observed.

Hierarchical clustering analysis based on the presence/absence of the adhesin, biofilm, and regulatory genes revealed 20 different clusters (Supplementary Table 1). One hundred and twenty-seven (26.5%) strains (13 clinical and 114 sub-clinical) of the 478 genomes evaluated lacked the 35 adhesion and biofilm-associated genes identified by the RAST annotation tool. The staphylococcal species lacking these genes included: *S. arlettae, S. equorum S. gallinarum S. sciuri, S. succinus, S. vitulinus* and *S. xylosus*. In species heatmaps (Fig. 3), the pattern of adhesion/biofilm genes in clinical isolates differed from that of sub-clinical isolates. The presence of the *clfA, clfB, fnbA, spa, sdrC, coa, eap, emp, vWbP, sasD, icaABCD* and *icaR* genes was highly correlated in clinical isolates, while in subclinical isolates, no specific gene correlations were observed (Spearman coefficient > 0.8). Based on hierarchical matrix clustering, clusters 9 and 10; 19 and 18 and 4 and 5 (Supplementary Table 1) contained most of the strains that harbored a typical pattern of nine genes (*rbf, pls, sasF, sarA, atl, sasH, sigB, tcaR* and *ebpS*) in both clinical and subclinical isolates. This pattern is also demonstrated in the heatmap of the gene frequency (Fig. 4).

## Discussion

*Staphylococcus* spp. are the most common etiologic agents of mastitis, with *S. aureus* thought the most important, while coagulase-negative staphylococci and non-aureus staphylococci considered less significant^6^. Based on 16S RNA identification of the 478 available genome sequences, *S. chromogenes* (28.7%) and *S. simulans* (20.0%) were the staphylococcal species most frequently associated with clinical mastitis. *S. aureus* was the next most prevalent species (18.7%) associated with clinical mastitis and it was rarely (3.3%) associated with subclinical mastitis deposits in GenBank. Most subclinical cases were associated with *S. chromogenes* (15.6%) followed by *S. simulans* (6.8%), *S. xylosus* (6.5%), *S. haemolyticus* (6.3%), *S. cohnii* (5.8%), *S. epidermidis* (5.5%), *S. capitis* (5.3%), *S. sciuri* (5.3%), *S. gallinarum* (5.0%), *S. warneri* (4.8%), *S. equorum* (4.5%), *S. saprophyticus* (4.0%), *S. succinus* (3.8%), *S. arlettae* (3.5%), and *S. agnetis* (3.3%). These findings are consistent with a growing number of studies which report that coagulase-negative staphylococci are emerging pathogens associated with mastitis and persistence of intramammary infection in bovine worldwide^7^. As observed in this study, in a recent Canadian study, the *S. chromogenes* and *S. simulans* were among the most common species found in clinical mastitis cases^8^.

Adherence is considered the first step of staphylococcal infection and the presence of biofilm aids in the process. Accordingly, adhesion-related genes are thought to be key virulence factors^8^. In the current study, the most frequently observed adherence and biofilm-forming genes were e*bpS, atl, pls, sasH, sasF, rbf, tcar, sarA* and *sigB* in both clinical and subclinical isolates. The genomes of some subclinical species i.e., *S . arlettae, S. succinus, S. sciuri, S. equorun, S. galinarum* and *S. saprophyticus* lacked most of the adherence and biofilm genes, which could indicate that these species are more likely to be contaminants associated with the milk microbiota^9,10^ rather than subclinical mastitis agents. Nevertheless, the *S. equorum. S. sciuri, S. galinarum*, and *S. succinus* isolates have been associated with skin and urinary tract infections in humans and mice^11–13^ which indicates that they can potentially harbor virulence genes.

These findings supports previous reports on the high presence of the *ebpS* gene in subclinical mastitis staphylococcal isolates from China, Iran and Poland^14–16^. The elevated incidence of this gene in these isolates was attributed to the fact that it mediate the binding to surface proteins or soluble elastin peptides on host cells, therefore it’s importance since cell-binding is the first step of staphylococcal infection^14^. The *atl* gene was the second most frequently detected gene. It encodes an autolytic protein that can cause the lysis of other bacterial that compete with *Staphylococcus* spp. for the acquisition of nutrients in the milk^17^. This gene is also associated with bacterial internalization and secretion of proteins in *S. aureus*^18^. This gene’s presence in most mastitis isolates could be attributed to the fact that it is implicated in diverse functions such as bacterial attachment to surface, lysis mediated biofilm formation and secretion of the cytoplasmic proteins from the staphylococcal cell wall. The *atl* gene has also been implicated in adherence to fibronectin, heparin, and gelatin^19^ which could confer an advantage during infection as heparin is released by mast cells and basophils at the site upon tissue damage^20^. The same could be noted about the *pls* gene, which encodes the plasmin-sensitive protein that has a role in adherence and is an important virulence factor in mouse septic arthritis model^4^. To date, and atl genes have not been well studied–even in recent genomic comparison studies of S. *aureus* isolates from bovine mastitis^21,22^.

The surface proteins encoded by *sasH* and *sasF*, play an essential role in virulence because they can bind to host extracellular matrix and plasma components. They have been recently reported as the most prevalent adhesins in a genome comparison study of 24 bovine-associated staphylococcal isolates, with all isolates positive for both genes^5^. The *sasH* gene is significantly associated with invasive disease isolates due to its ability to inhibit the oxidative burst and promote *S. aureus* survival in neutrophils^20^ thus allowing the organism to avoid the bovine immune response and colonize the mammary gland. In the current study, the *sasH* gene was not only present in *S. aureus*, but was also detected in *S. chromogenes, S. haemolyticus, S. simulans, S. agnetis, S. capitis,* and *S. warneri*. In contrast, Little is known about *sasF* and its role in virulence but it is believed that it may have an important role in thromboembolic lesions^23^ and in advanced stages of mastitis when capillary damage caused by *S. aureus*^24^.

The *coa*, *eap*, *emp efb* and *vWbp* genes were most frequently present in clinical mastitis isolates and their presence was highly correlated with the presence of the *icaABCD* and R genes. This correlation was not observed in subclinical isolates. The presence of coagulase is commonly associated with virulence since it is known that *coa* positive strains are more resistant to neutrophil activities than those which lack the gene^25^. Also, the *vWbp* is another known coagulase in *Staphylococcus* likely has a similar effect^26^. The detection of the *eap* gene was only recently describe in strains of *S. aureus* from subclinical mastitis cases in china^14^. Manual examination of the *S. aureus* SAMN02603524 (NC_021670.1) genome revealed that the *emp* gene is located 300 nucleotides downstream from the *vWbp* gene (Supplementary Fig. 14), but no other close spatial relationships were observed with the other genes. Although the *eap*, *emp* and *vWbp* do not share a common promoter, there is evidence that their expression is regulated by a conserved octanucleotide sequence (COS) and since they are involved in modulating the immune response to *S. aureus* infections or antibiotic, it is possible to assume that the *emp* gene would work with the *vWbp* gene in *S. aureus* immune response evasion^27,28^. The *eap* gene product has recently been shown to suppress the formation of “neutrophil extracellular traps” (NETs), which are thought to function as a neutrophil-mediated extracellular trapping mechanism^29^.

The *icaABCD* operon is the most studied *Staphylococcus* biofilm forming genes and it is most frequently reported in mastitis isolates highlighting their potential to form biofilm^30^. In the current study, the *icaC* gene was the most prevalent in clinical and subclinical isolates, in contrast to a previous report of which *icaA* and *icaD* are the most prevalent^31^. Also, the finding of this study observed that coagulase negative *Staphylococcus* (CoNS) and *S. aureus* possessed the *icaA* and/or *icaD* gene in contrast with a previous findings that the *icaA* was only observed in CoNS strain while the *icaD* was found both in CoNS and *S. aureus*. The most prevalent biofilm regulatory genes detected were: *rbf*, *tcaR*, *sarA* and *sigB* which is in agreement with previous finding of high presence of *sarA*, *tcaR* in S.aureus from bovine subclinical mastitis isolates^32^. The *rbf* gene is an important biofilm regulatory gene and its inactivation results in a biofilm negative phenotype^33^. It has recently been shown that *rbf* mutants exhibit significantly increased pathogenicity compared to the wild type *S. aureus* strains^34^ suggesting an important role in host adaptation. The *rbf* gene product negatively regulates hemolytic activity by repressing the expression of the *hla* and *psmA* genes. It also upregulates *sarX,* which, in turn, activates the *icaADBC* locus leading to biofilm production^35^.

The *tcaR* gene increases the production of PIA by regulating the expression of the *icaADBC* operon and the *spa*, *sasF* and *sarS* genes^35^. Given the high frequency of the *sasF* gene observed in this study, detection of its transcriptional regulators was not unexpected. The *sarA* family of transcriptions regulators proteins are responsible for controlling many target genes involved in virulence. Most notably, SarA is responsible for regulating the *agr* loci, which is a pivotal regulator of virulence in *S. aureus*^36^. The presence of the *sarA* gene in mastitis was observed in a recent study int all of the 84 *S. aureus* isolates from mastitis cases in Xinjiang, China^36^. The rRNA polymerase sigma factor (SigB) has a central role in stress homeostasis. This protein contributes to the synthesis of several virulence determinants defining staphylococcal pathogenesis, including the transcriptional activation of many surface proteins (such as *clf*A and *fnb*A) while downregulating the production of secreted toxins and proteases (such as Aur, SspA, SspB)^37^.

Phylogenetic analysis of *Staphylococcus* spp. related to mastitis has been done before, studies to date have focused on comparatively few isolates and mainly on *S. aureus* and observed that strains that had different origin were clustered togeter^38^*.*In this study, phylogenetic analysis of the 16S RNA genes indicated that *S. aureus, S. epidermidis, S.caprae* and *S. capitis* have a close relationship as observed in human clinical isolates^39^. Also, these species all possessed and shared a large number of adhesion genes. In a previous study, some authors have suggested that dairy cows can be subclinically infected with *S. aureus* subtypes that can cause clinical mastitis if the right conditions are present^38^. In the current study, some clinical and subclinical strains clustered together based on their16S RNA sequences, but they had different biofilm and coagulase gene contents. Also, this study shows that *S. chromogenes* isolates from cases of clinical and subclinical mastitis were closely related to *S. agnetes* and *S. hyicus* suggesting that these species could also demonstrated the same potential to became an emerging mastitis agent as *S. chromogenes*^40^.

*S. aureus* is reported to acquire and disseminate SaPIs through HGT events mediated by phages^41^. Moreover, *S. aureus* colonization of different host species is known to be facilitated by the HGT of virulence factors across different staphylococcal species^42^. It is further known that biofilm growth can increase the rate of HGT of virulence determinants such as antibiotic resistance genes^43^. In this study, the co-phylogenetic analysis suggested that HGT amongst clinical and subclinical isolates of *S. chromogenes*, *S. aureus*, and *S. simulans* (mainly *ebpS*, *rbp*, *sarA*, *tcaR*, *pls)* and *sigB* gene in *S. aureus* may occur. Moreover, the phylogenetic relationship of the adhesion and biofilm genes: *ebpS, sasH, atl, sar*A*, rbf* and *tca*R are different from 16S phylogenetic distribution. This finding is consistent with the notion that HGT occurs among clinical and subclinical isolates^44^. It is therefore tempting to speculate that virulence factors may arise in staphylococcal species not generally associated with clinical mastitis by known *Staphylococcus* HGT mechanisms, but further study is need to demonstrate this.

Although the arbitrary source of isolates used (478 staphylococcal spp. isolated from clinical and subclinical mastitis from Brazil, Canada, India, Netherlands, and United States which had sequenced) might have introduced some biases, a number of the adhesins, biofilm, and related regulatory genes identified in this study might be useful for virulence profiling or as targets for vaccine development for mastitis-related staphylococcus species.

## Methods

### Genomic data

The genomes of *Staphylococcus* spp. from clinical (n = 80) and subclinical (n = 398) mastitis cases worldwide were downloaded from the National Center for Biotechnology Information (NCBI). An initial advanced search of the NCBI Biosample database I with “mastitis” and “staphylococcus” as keywords resulted in 925 entries. After this initial step, only complete genomes that were identified as mastitis isolates from *Bos taurus* or *Bubalus bubalis* and which were the sole agent associated with the diseases were evaluated. Also, for more detailed information, the publications or their BioSample descriptions were evaluated (Supplementary Table 1). It was assumed that mastitis states were classified according to the clinical presentation and standard triage test described by Radostits et al.^3^. Genomes in this study were from bacteria isolated in Brazil^45^, Canada^46^ India, Netherlands, and United States (information regarding isolates BioSample available on Supplementary Table 1).

### Genome annotation and adhesion-related gene identification

Genomes were annotated using Rapid Annotation using Subsystem Technology (RAST)^47,48^. The sequences of the genes classified as adhesion/adhesins or implicated in biofilm formation and their respective regulatory genes were downloaded and analyzed manually. The genes were considered to encode adhesins or be play a role in biofilm formation based on their classification in the VFDB reference database for bacterial virulence factors^49^ and/or in the RAST annotation engine^47^. 16S rRNA gene sequences were obtained from the complete genomes using the Basic Rapid Ribosomal RNA Predictor (Barrnap) v 0.9 (https://github.com/tseemann/barrnap).

### Data analysis

The presence or absence of selected genes was used in hierarchical clustering analysis with PAST software v4.03^50^. Clusters of the isolates were created based on the most and least frequent genes. The Spearman test was used to analyze the correlation between the presence/absence of adhesin and biofilm genes in both clinical and subclinical mastitis isolates (A coefficient close to 1.0 indicates a high correlation). Gene profiling by frequency heatmaps was calculated using Numpy v1.20.3^51^. Graphs were made using Matplotlib v3.4.2^52^ and, when needed, with R-software v4.1.0^53^. The statistical significance of gene presence and mastitis state was obtained by logistic regression with R software.

### Phylogenetic analysis and tree construction

The phylogenetic correlation of the 16S RNA, *araC*, *pls*, *sasF*, *sarA*, *atl*, *sasH*, *sigB tcaR*, and *ebpS* genes was determined and phylogenetic trees were generated with maximum likelihood approach using IQ-TREE2 from MAFFT v7^54^ gene aligment using defalt parameter. For clade support, we performed utrafast bootsrap analses with 1000 pseudoreplicates implemented in IQ-tree^55^. Tree visualization was done with iTOL v5^56^. The co-phylogenetic tree construction was done using phytools v0.7-80 in R-software v4.1.0^53^. In order to create a rooted tree, the closely related *Escherichia coli* strains (accession numbers): 2014C-3057 (NZ_CP027387.1); 2015C-4944 (NZ_CP027390.1); 2013C-4538 (NZ_CP027582.1); E2865 (NZ_AP018808.1); 97–3250 (NZ_JHEW00000000.1); FORC_028 (NZ_CP012693.1); 2013C-4225 (NZ_CP027577.1); 2014C-3050 (NZ_CP027472.1); 2012C-4606 (NZ_CP027352.1) and CFSAN027343 (NZ_CP037943.1) were used as an outgroup.

## Supplementary Information

Supplementary Information 1.

Supplementary Information 2.

Supplementary Information 3.

Supplementary Information 4.

Supplementary Information 5.

Supplementary Information 6.

Supplementary Information 7.

Supplementary Information 8.

Supplementary Information 9.

Supplementary Information 10.

Supplementary Information 11.

Supplementary Information 12.

Supplementary Information 13.

Supplementary Information 14.

Supplementary Information 15.
