# Supplementary material for: Genomic comparisons and phylogenetic analysis of mastitis-related staphylococci with a focus on adhesion, biofilm, and related regulatory genes
Source: Sci Rep. 2021 Aug 30;11:17392. doi: 10.1038/s41598-021-96842-2 (PMC8405628; doi:10.1038/s41598-021-96842-2)
Supplement: Supplementary file 5 — Supplementary Information 5. [file 41598_2021_96842_MOESM5_ESM.pdf]

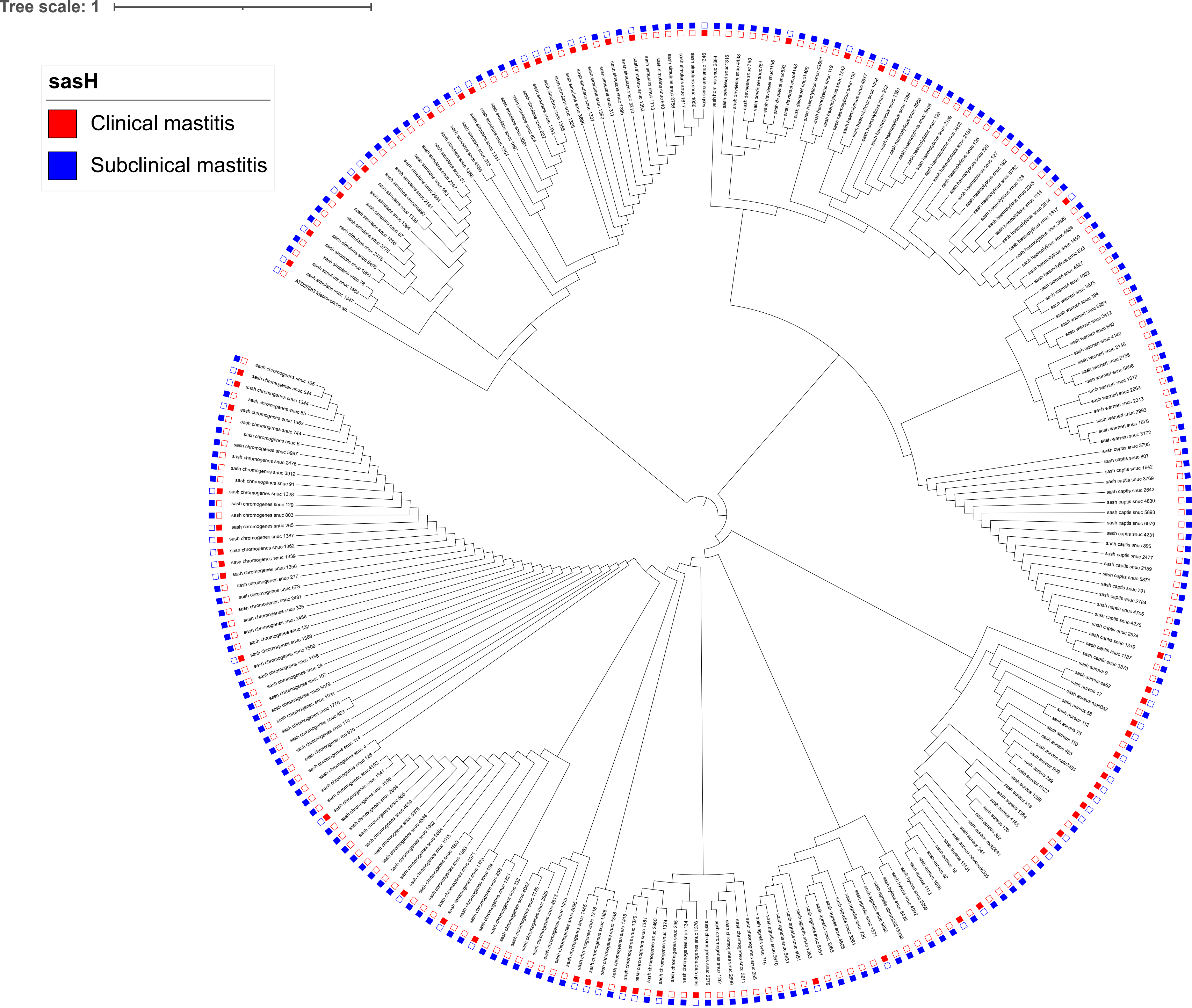

Supplementary Figure 5. Phylogenetic *sasH* gene tree of staphylococcal species associated with clinical and subclinical mastitis
